# P02.135. Measuring mindfulness: which aspects of mindfulness change following a brief telehealth intervention for PTSD?

**DOI:** 10.1186/1472-6882-12-S1-P191

**Published:** 2012-06-12

**Authors:** B Niles, A Seligowski, A Silberbogen

**Affiliations:** 1 National Center for PTSD at VA Boston Healthcare System, Boston, USA; 2 VA Boston Healthcare System and Boston University School of Medicine, Boston, USA

## Purpose

The goal of this pilot study was to examine changes in self-reported levels of mindfulness in the context of an 8-week mindfulness telehealth intervention for military veterans with combat-related posttraumatic stress disorder (PTSD).

## Methods

Participants were 24 male combat veterans with PTSD aged 23 to 66 (M=55.2). Participants were randomized to either a mindfulness telehealth intervention or a PTSD psychoeducation telehealth condition. Both interventions consisted of two in-person and six telephone individual sessions. Measures were collected pre- and post-treatment and at 6-week follow-up and included the Mindful Attention Awareness Scale (MAAS), the White Bear Suppression Inventory (WBSI), and the Five Facets of Mindfulness Questionnaire (FFMQ).

## Results

Significant condition by time interaction effects were found in the expected direction for the WBSI (p=.04) and FFMQ Describing facet (p=.02) and trends were detected for the MAAS (p = .07) and the FFMQ Observing facet (p = .07), indicating gains in skills for the mindfulness group over the course of the study. Results also suggest that a brief telehealth intervention introducing mindfulness skills may be effective in increasing levels of mindfulness as measured by these instruments.

## Conclusion

This study represents one of the few published studies to use multiple measures of mindfulness to examine changes in mindfulness skills and the first known published study to examine changes in mindfulness following a brief telehealth intervention. Limitations include a small sample size and lack of intent-to-treat analyses. A short treatment that does not require group participation and that can be easily accessed may be especially appealing to more reluctant participants, such as those with PTSD or other psychological disorders. Identifying the aspects of mindfulness that are sensitive to change represents a first step in determining whether adoption of mindfulness skills may be an important mechanism of action.

